# Dissecting the roles of EIF4G homologs reveals DAP5 as a modifier of CGG repeat-associated toxicity in a *Drosophila* model of FXTAS

**DOI:** 10.1016/j.nbd.2023.106212

**Published:** 2023-06-22

**Authors:** Indranil Malik, Yi-Ju Tseng, Clare M. Wieland, Katelyn M. Green, Kristina Zheng, Katyanne Calleja, Peter K. Todd

**Affiliations:** a Department of Neurology, University of Michigan, Ann Arbor, MI, USA; b Cellular and Molecular Biology Graduate Program, University of Michigan, Ann Arbor, MI, USA; c Neuroscience Graduate Program, University of Michigan, Ann Arbor, MI, USA; d Ann Arbor Veterans Administration Healthcare, Ann Arbor, MI, USA

**Keywords:** Fragile X, FXTAS, RAN translation, DAP5, repeat expansion disorders

## Abstract

Neurodegeneration in Fragile X-associated tremor/ataxia syndrome (FXTAS) is caused by a CGG trinucleotide repeat expansion in the 5′ UTR of *FMR1*. Expanded CGG repeat RNAs form stable secondary structures, which in turn support repeat-associated non-AUG (RAN) translation to produce toxic peptides. The parameters that impact RAN translation initiation efficiency are not well understood. Here we used a *Drosophila melanogaster* model of FXTAS to evaluate the role of the eIF4G family of eukaryotic translation initiation factors (EIF4G1, EIF4GII and EIF4G2/DAP5) in modulating RAN translation and CGG repeat-associated toxicity. DAP5 knockdown robustly suppressed CGG repeat-associated toxicity and inhibited RAN translation. Furthermore, knockdown of initiation factors that preferentially associate with DAP5 (such as EIF2β, EIF3F and EIF3G) also selectively suppressed CGG repeat-induced eye degeneration. In mammalian cellular reporter assays, DAP5 knockdown exhibited modest and cell-type specific effects on RAN translation. Taken together, these data support a role for DAP5 in CGG repeat associated toxicity possibly through modulation of RAN translation.

## 1. Introduction

More than 50 different human diseases result from pathogenic expansions of short tandem repeats (Malik et al., 2021a). These repeat expansions preferentially impact the nervous system, leading to neurodegenerative and neurodevelopmental disorders. Fragile X-associated tremor/ataxia syndrome (FXTAS) is a progressive and fatal adult-onset neurodegenerative disorder that afflicts approximately 1/5000 people (Jacquemont et al., 2004; Tassone et al., 2012). FXTAS is caused by a pathogenic trinucleotide CGG repeat expansion in the 5’ UTR of the Fragile X Messenger Ribonucleoprotein gene (*FMR1*) (Hagerman and Hagerman, 2015; Hagerman et al., 2001). Normally the *FMR1* 5’ UTR harbors less than 30 CGG repeats, which acts to regulate Fragile X Messenger Ribonucleoprotein (FMRP) expression in neurons (Rodriguez et al., 2020). In FXTAS, CGG repeats expanded to 55–200 in number, leading to neurological symptoms that include dementia, imbalance, and tremors (Hagerman and Hagerman, 2015; Jacquemont et al., 2003). FXTAS pathology includes accumulation of ubiquitinated and p62-positive intranuclear neuronal inclusions in the brains along with widespread neuronal loss and brain atrophy (Ariza et al., 2016; Greco et al., 2006, 2002; Willemsen et al., 2003). Despite intensive research efforts, there are currently no disease modifying therapies for FXTAS or related repeat expansion disorders.

CGG repeat expansions in FXTAS elicit toxicity through at least two RNA-centric mechanisms. The expanded repeat RNAs can aberrantly interact with RNA-binding proteins (RBPs) and sequester them to form large RNA–protein complexes that can appear as nuclear foci (Glineburg et al., 2018; Jin et al., 2007, 2003; Tassone et al., 2004). Studies involving *in vitro* RNA pull-down assays or *in vivo* RNA capture assays identified multiple RBPs including Pur alpha, hnRNP A2/B1, Sam68, DROSHA/DGCR8 and serine/arginine-rich splicing factors (SRSFs) as potential interactors of expanded CGG repeat RNA (Jin et al., 2007; Malik et al., 2021b; Sellier et al., 2013, 2010; Sofola et al., 2007). Overexpression of some of these factors suppress CGG repeat toxicity in a *Drosophila melanogaster* (fly) model of FXTAS, suggesting functional loss of sequestered proteins may contribute to repeat pathogenesis (Jin et al., 2007; Sofola et al., 2007). Expanded repeat RNAs also support a non-canonical form of translation in the absence of AUG start codons known as repeat-associated non-AUG (RAN) translation (Todd et al., 2013; Zu et al., 2011). In FXTAS, RAN translation can occur in multiple reading frames and from anti-sense transcripts to produce homopolymeric repeat proteins (Kearse et al., 2016; Krans et al., 2016; Todd et al., 2013). RAN translation in the +1 reading frame (GGC repeat) generates a polyglycine-containing peptide termed FMRpolyG, which is the most abundant RAN translation product (Buijsen et al., 2014; Todd et al., 2013). In both patients and model systems, FMRpolyG accumulates within neuronal inclusions, and exogenous expression of CGG repeat RNA depends on FMRpolyG production to elicit toxicity in human cells, rodent neurons, flies, and transgenic mice (Sellier et al., 2017; Todd et al., 2013).

In FXTAS, RAN translation initiates predominantly at near-AUG codons present upstream of CGG repeats through a mechanism that is analogous to the canonical translation initiation process (Kearse et al., 2016). Canonical translation initiation begins with the recognition and binding of the 5′-m^7^G cap of a mature mRNA with the EIF4F complex, which is consisting of the cap-binding factor EIF4E, DEAD-box RNA helicase EIF4A and the scaffold protein EIF4G (Green et al., 2016; Jackson et al., 2010). Subsequently, the 43S pre-initiation complex (PIC), containing the 40S small ribosomal subunit, the EIF2⋅GTP⋅Met-tRNAi ternary complex, and other initiation factors including EIF1, EIF3, and EIF5, joins the EIF4F complex (Green et al., 2016; Jackson et al., 2010). The fully assembled PIC scans the 5’ UTR in search of an AUG start codon in optimal sequence context to initiate translation. We previously discovered that, like canonical translation, RAN translation initiation at CGG repeats uses a cap- and eIF4A-dependent scanning mechanism (Kearse et al., 2016). However, RAN translational initiation on CGG repeats also occurs within the repeat and in the absence of any near-cognate codons (Kearse et al., 2016; Zhang et al., 2022), and at Amyotrophic lateral sclerosis/ frontotemporal dementia (ALS/FTD)-associated GGGGCC repeats, RAN translation can occur through cap-independent mechanisms (Cheng et al., 2018; Wang et al., 2021). These findings suggest that the exact mode of RAN translation initiation is dependent on the repeat sequence, the repeat context within the RNA and the surrounding sequence. In addition, both cell state and cell type can influence the efficiency of RAN translation in certain contexts (Cheng et al., 2018; Green et al., 2017; Westergard et al., 2019; Zu et al., 2011).

Determining how RAN translation initiation at CGG repeats differs from canonical translation initiation might reveal new therapeutic targets for FXTAS. Since these two modes of initiation considerably overlap in mechanism, interrogating factors involved in each step of the initiation process can precisely dissect how their roles may vary between these two processes. Previously, we have used a *Drosophila* model of FXTAS to identify translation initiation factors that may preferentially promote RAN translation initiation at CGG repeats (Linsalata et al., 2019). Here we used this same FXTAS fly model to dissect the role of EIF4G homologs in modulating RAN translation and CGG repeat-associated toxicities. Most eukaryotes contain three homologs of EIF4G, termed EIF4G1, EIF4GII and EIF4G2 (also known as DAP5 and Nat1) (Das and Das, 2016). While EIF4G1 and EIF4G II are structurally very similar, DAP5 is slightly different as it does not contain EIF4E and PABP binding domains (Imataka et al., 1997; Levy-Strumpf et al., 1997; Yamanaka et al., 1997). For this reason, DAP5 was initially implicated in cap-independent translation initiation, where it has specific roles in regulating cellular transcripts that utilize internal ribosome entry site (IRES)-dependent translation initiation such as p53, Apaf-1 and Bcl-2 (Henis-Korenblit et al., 2002; Liberman et al., 2015; Marash et al., 2008; Marash and Kimchi, 2005). However, DAP5 is also required for cap-dependent translation of a wide range of transcripts (de la Parra et al., 2018; Volta et al., 2021). Importantly, DAP5-deficiency does not markedly impact bulk translation, but can affect specific cellular functions such as cell proliferation and stem cell differentiation (Lee and McCormick, 2006; Sugiyama et al., 2017; Yamanaka et al., 2000; Yoffe et al., 2016).

Here we show that selective loss of DAP5 or DAP5-interacting initiation factors (EIF2β, EIF3F and EIF3G) strongly suppresses CGG repeat-associated toxicity in a fly model of FXTAS. Genetic ablation of DAP5 suppressed severe eye degeneration elicited by expression of CGG repeats in the fly eye. Furthermore, DAP5 knockdown of CGG repeat-expressing flies led to a marked reduction in the accumulation of RAN translated products. Using cellular reporters, we show that DAP5 has cell-type specific effects on RAN translation. Together, these data provide insight into the roles of EIF4G homologs in RAN translation at CGG repeats that may contribute to neurodegeneration.

## 2. Results

### 2.1. Identification of DAP5 as a strong modifier of CGG repeat-associated toxicity in a Drosophila model of FXTAS

**Fig. 1.**
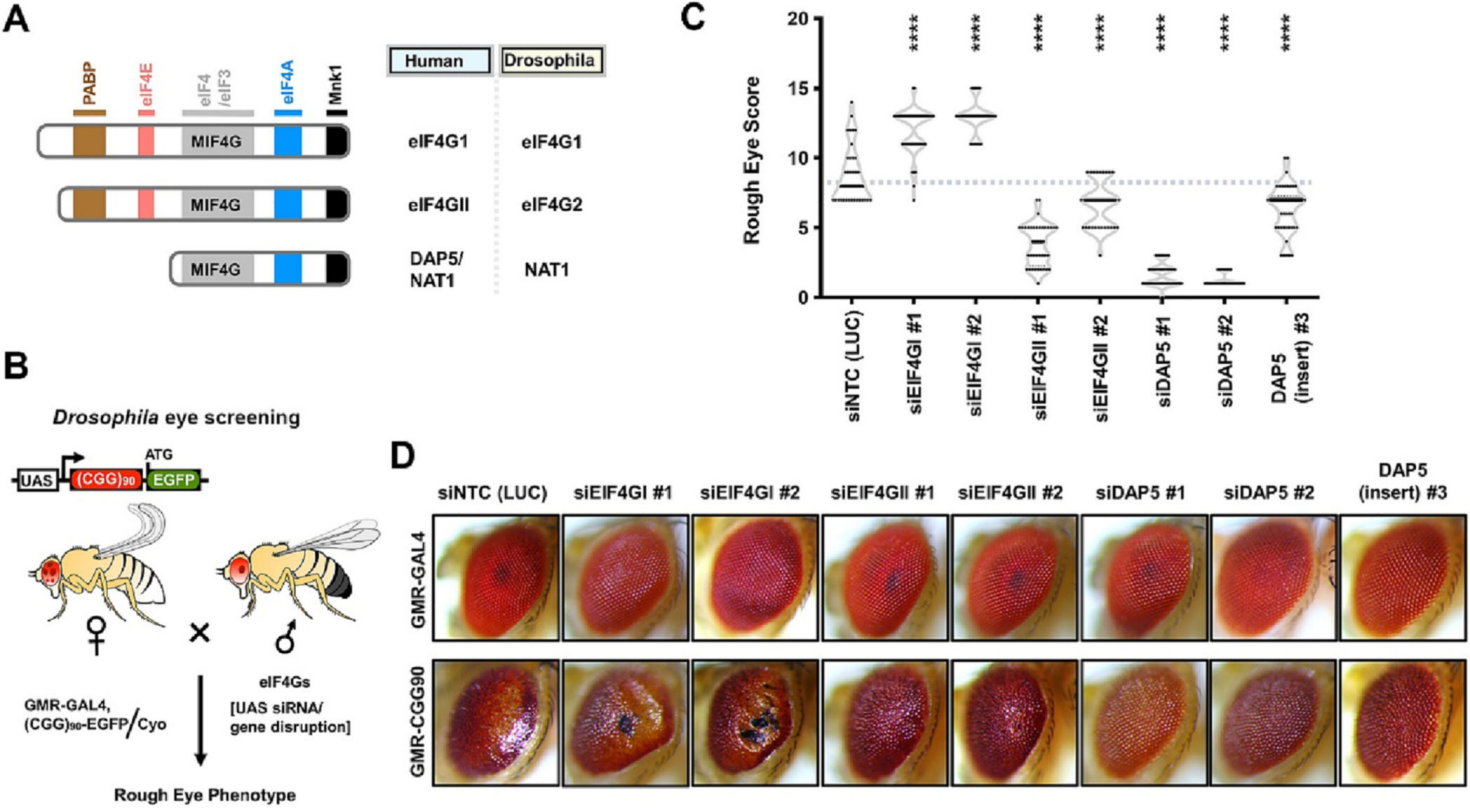
Genetic ablation of DAP5 suppresses CGG repeat expansion-associated toxicity in a *Drosophila* model of FXTAS. (A) Domain architecture of eIF4Gs with their nomenclatures in human and *Drosophila*. Color-coded domains highlight binding/interacting regions for respective factors. (B) A schematic of *Drosophila* screening to evaluate the role of eIF4Gs in modulating rough eye phenotype caused by CGG repeat expansion. (C) Quantitation of uas-(CGG)90-EGFP eye phenotype with eIF4G modifiers. siNTC = non-targeting siRNA against luciferase (LUC) gene. Different siRNA/insertion lines for the same target are numbered (#1, #2 and #3). Violin plots show all rough eye scores for *n* ≥ 30 flies/genotype with solid lines representing the median value. One-way ANOVA with Dunnett’s multiple comparison test, **** *P* < 0.0001. (D) Representative pictures of fly eyes expressing uas-(CGG)90-EGFP under a GMR-GAL4 driver, with disruptions of various eIF4Gs.

To identify potential suppressors of CGG repeat expansion-associated cellular toxicity underlying FXTAS, we conducted a candidate-based modifier screen using a *Drosophila* model of FXTAS. Expression of an expanded CGG repeat harboring transgene in the *Drosophila* eyes results in a degenerative (rough eye) phenotype, which enables screening of modifier genes that might suppress or enhance rough eye phenotype (Jin et al., 2007; Todd et al., 2010). We used this fly model of FXTAS earlier to discover several non-canonical translation initiation factors and RNA-binding proteins (RBPs) that specifically modulate CGG RAN translation and, in general, suppress CGG repeat expansion-associated toxicity (Green et al., 2022; Linsalata et al., 2019; Malik et al., 2021b). In this study, we evaluated the role of EIF4G family proteins in modulating toxic phenotypes of CGG repeat expansion in a fly model of FXTAS (Supplementary Fig. 1 and Fig. 1A–B). To this end, we used *Drosophila* orthologs of human EIF4Gs to cross with flies expressing (CGG)90-EGFP transgene under a GMR-GAL4 driver (Fig. 1B). We examined eye phenotypes of the F1 flies to identify suppressors and/or enhancers of (CGG) 90-driven toxicity. In parallel, modifier lines were crossed to flies carrying a GMR-GAL4 driver alone to evaluate the effects of knockdown independent of (CGG)90 expression (Fig. 1C–D).

We observed that genetic ablation of EIF4G1 resulted in significant enhancement of rough eye phenotype as manifested by severe eye degeneration and the presence of necrotic tissues in the eyes (Fig. 1C–D). Disruption EIF4G1 alone did not show significant changes in the external eye appearance of control GMR-GAL4 flies, indicating that the enhancement of rough eye phenotype is specific to the (CGG)90-expressing flies. Interestingly, knockdown of other two EIF4G homologs EIF4G II and DAP5 (fly NAT1) led to suppression of rough eye phenotypes. While the knockdown of EF4G II led to a modest suppression of (CGG)90-based eye toxicity, knockdown of DAP5 resulted in significant rescue of eye degeneration to a near-wild type state across multiple fly lines (Fig. 1C–D). Together these results indicate that the EIF4G family of proteins modulates CGG repeat elicited toxicity in the fly model of FXTAS.

### 2.2. EIF2β modifies CGG repeat-associated toxicity in a Drosophila model of FXTAS

DAP5 differs from other eIF4G homologs in that it lacks EIF4E and PABP-binding domains (Supplementary Fig. 1 and Fig. 1A). As such, DAP5 exhibits a distinct initiation factor and RNA-binding protein interactome compared to other eIF4G homologs (de la Parra et al., 2018; Sugiyama et al., 2017). In reviewing prior studies, we observed that multiple EIF2 and EIF3 subunits interact selectively with DAP5 (Supplementary Fig. 2A–B). We, therefore, assessed whether the loss of DAP5-specific interaction partners might mimic the DAP5 loss of function phenotype and modify CGG repeat-associated toxicity in flies.

**Fig. 2.**
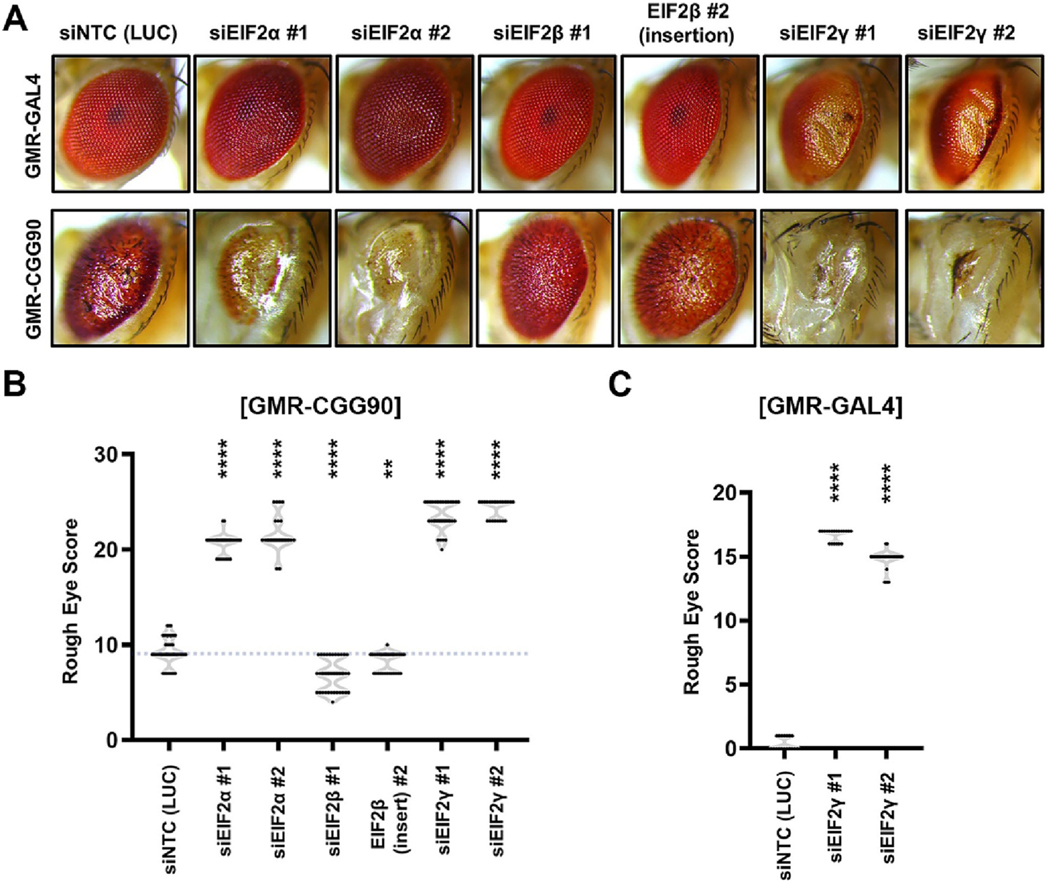
Genetic ablation of eIF2β suppresses CGG repeat expansion-associated toxicity in *Drosophila*. (A) Representative pictures of fly eyes expressing uas-(CGG)90-EGFP under a GMR-GAL4 driver, with eIF2 subunit modifiers. (B) Quantitation of rough eye scores (n ≥ 30 flies/genotype) of flies expressing uas-(CGG)90-EGFP. siNTC = non-targeting siRNA against luciferase (LUC) gene. (C) Quantitation of rough eye phenotypes (n ≥ 30 flies/genotype) induced by siRNAs against eIF2γ alone in absence of uas-(CGG)90-EGFP compared to control siRNA. Statistical test for (B-C): One-way ANOVA with Dunnett’s multiple comparison test, **P < 0.01, **** P < 0.0001.

We first assessed whether knockdown of any of the 3 subunits of EIF2 which preferentially interact with DAP5, namely EIF2 α, β and γ, altered CGG repeat-associated toxicity in flies. Knockdown of eIF2 α and γ led to a severe enhancement of rough eye phenotype in (CGG)90-expressing flies (Fig. 2A–B). Knockdown of eIF2γ showed a more severe phenotype compared to eIF2α knockdown. We wondered if the enhancement of rough eye phenotypes caused by siRNA knockdown alone independent of (CGG)90 repeat expression. While knockdown of EIF2α independently did not show any severe phenotype compared to control siRNA, knockdown eIF2γ showed significant toxicity in GMR-GAL4 expressing flies (Fig. 2C). These results explain the aggravated rough phenotype of eIF2γ knockdown compared to eIF2α. Unlike knockdown eIF2α and γ, eIF2β knockdown significantly suppressed the rough eye phenotype in (CGG)90-expressing flies (Fig. 2A–B). Interestingly, knockdown of eIF2β did not show any toxic phenotype in GMR-GAL4 expressing flies alone (Fig. 2A). This result is consistent with an earlier observation that DAP5 associates with eIF2β to promote non-canonical translation initiation (Liberman et al., 2015).

### 2.3. EIF3F and EIF3G strongly suppress CGG repeat-associated toxicity in Drosophila

**Fig. 3.**
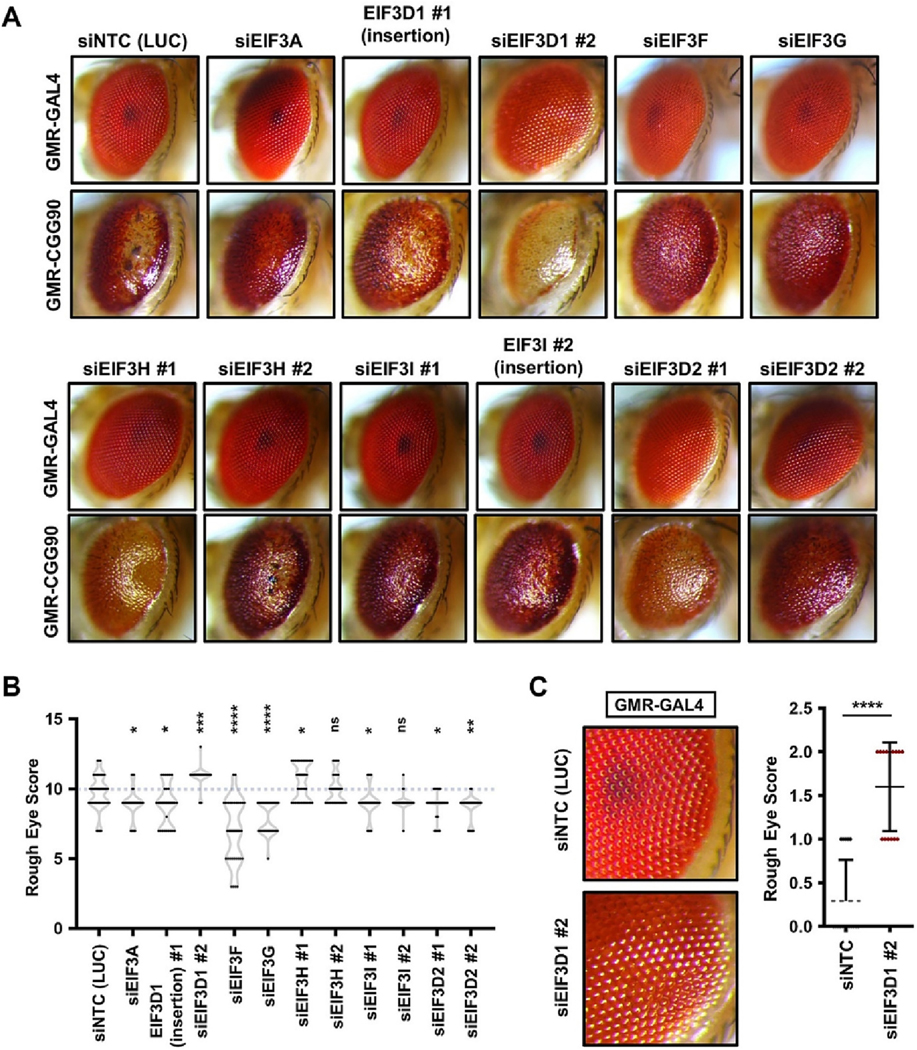
Knockdown of select eIF3 subunits suppress CGG repeat-associated toxicity in *Drosophila*. (A) Representative pictures of fly eyes expressing uas-(CGG)90-EGFP under a GMR-GAL4 driver, with knockdown of selective eIF3 subunits. (B) Quantitation of rough eye scores (*n* ≥ 25 flies/genotype) of flies expressing uas-(CGG)90-EGFP. siNTC = non-targeting siRNA against luciferase (LUC) gene. (C) Quantitation of rough eye phenotypes (n ≥ 30 flies/genotype) induced by siRNAs against *Drosophila* eIF3D1 alone under a GMR-GAL4 driver. Expanded views of a section of fly eye picture (from panel A) is presented for comparison. Statistical test for (B-C): One-way ANOVA with Dunnett’s multiple comparison test, * *P* < 0.05, ** *P* < 0.01, *** *P* < 0.001, **** *P* < 0.0001 and ns = non-significant.

We next evaluated a possible role for EIF3 subunits that are enriched in the DAP5-interactome as potential modulators of CGG repeat-associated toxicity. In a previous study, knockdown of EIF3B exacerbated CGG repeat toxicity in flies (Linsalata et al., 2019). In this study, we assessed the effect of knocking down EIF3A, 3D, 3F, 3G, 3H and 3I. Most of the EIF3 subunits tested showed modest or no effect on the CGG repeat elicited rough eye phenotype. Interestingly, EIF3F and EIF3G knockdown showed strong suppression of the CGG repeat-associated rough eye phenotype (Fig. 3A–B). EIF3G has been shown to directly interact with CGG repeat expansion RNA in a prior study (Malik et al., 2021b). EIF3F has been previously implicated in Spinocerebellar ataxia type 8 (SCA8) associated RAN translation of polySer products from CAG repeats (Ayhan et al., 2018). Therefore, our observation is consistent with a possible role of EIF3F in CGG repeat RNA translation.

We also assessed the impact of lowering expression of EIF3D, which recognizes and binds the 5′-m^7^G cap of some mRNAs (Lamper et al., 2020; Lee et al., 2016). EIF3D interacts with DAP5 and modulates translation of a shared group of transcripts (de la Parra et al., 2018). However, knockdown of the *Drosophila* ortholog of human EIF3D1 showed inconsistent results (Fig. 3A–B). While one siRNA line displayed a modest suppression of the CGG repeat phenotype, a second line triggered a significant enhancement of the rough eye phenotype (Fig. 3A–B). This mixed effect may reflect the basal impact of knocking down EIF3D1, which led to modest toxicity in GMR-GAL4 flies lacking the CGG repeat (Fig. 3C). *Drosophila melanogaster* has a second closely related ortholog of human EIF3D1, termed EIF3D2. Interestingly, knockdown of EIF3D2 triggered a modest suppression of the CGG repeat-associated rough eye phenotype, without eliciting any significant toxicity in GMR-GAL4 expressing flies alone (Fig. 3A–B).

### 2.4. Knockdown of DAP5 inhibits + 1CGG RAN translation in (CGG)90-expressing flies

**Fig. 4.**
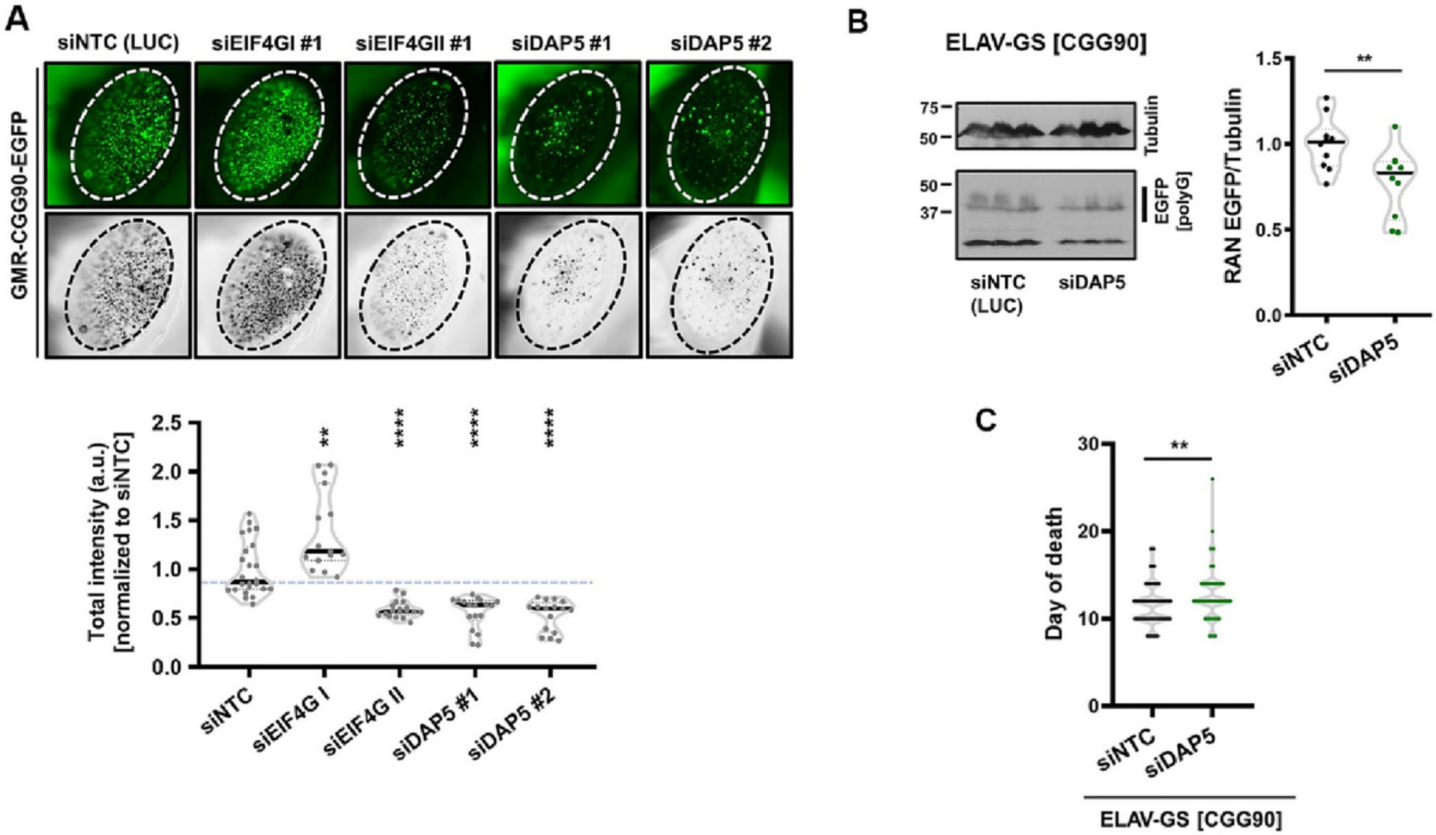
DAP5 knockdown reduces RAN translation product accumulation in *Drosophila.* (A) Representative photographs of *Drosophila* external eye showing accumulation of GFP aggregates caused by (CGG)90-EGFP transgene expression (left). Converted images used to quantify total intensity of GFP puncta (right). (B) Western blots of the FMRpolyG-EGFP RAN translation product in ELAV-GS driven (CGG)90-EGFP transgene expressing flies with and without DAP5 knockdown. Total protein from three (*n* = 3 biological repeats) independent fly crosses run in three replicates per gel (technical replicates). All data points presented in the graphs. (C) Survival data presented as violin plots showing the distributions of day of death events for each modifier (*n* = 120–140/genotype) after activation post-eclosion. Statistical analysis: (A) One-way ANOVA with Dunnett’s multiple comparison test; (B, C) Two-tailed Student’s *t*-test with Welch’s correction, ** P < 0.01, *** P < 0.001, **** P < 0.0001.

As DAP5 is an initiation factor, we hypothesized that the observed strong suppression of the CGG repeat triggered rough eye phenotype in the setting of lowered DAP5 expression might occur due to lower production of RAN translated products from CGG repeats in *Drosophila*. We previously observed that FMRpolyG accumulates within ubiquitin-positive aggregates within the fly eye (Todd et al., 2013) and that many (but not all) modifiers of CGG repeat expansion toxicity in flies also alter the accumulation of these inclusions (Green et al., 2022; Malik et al., 2021b). We, therefore, asked if any of the EIF4G modifiers alter the accumulation of GFP inclusions (from FMRpolyG-EGFP) in fly eyes. We observed that the knockdown of EIF4G1 enhanced GFP puncta accumulation and, conversely, the knockdown of both EIF4G II and DAP5 suppressed GFP puncta significantly (Fig. 4A). These changes in FMRpolyG-EGFP inclusions positively correlated with the modulations of eye phenotypes. Ubiquitous expression of the (CGG)90-EGFP transgene in the fly neurons under a gene-switch ELAV driver results in decreased survival of flies (Linsalata et al., 2019; Malik et al., 2021b). This decrease in lifespan results from the accumulation of RAN translated products pan-neuronally. Therefore, we asked if DAP5 knockdown has any effects on the accumulation of RAN translated products in flies expressing (CGG)90-EGFP transgene. Indeed, the knockdown of DAP5 led to a decrease in FMRpolyG-EGFP levels in adult flies as measured by immunoblot (Fig. 4B).

The Gene-switch system allows for selective UAS-transgene activation in flies post-eclosion based on exposure to a chemical activator (RU486). Expression of the (CGG)90-EGFP transgene in fly neurons under a gene-switch ELAV driver results in decreased survival of flies (Malik et al., 2021b) Mitigating RAN translation from CGG repeats enhances survival in flies (Linsalata et al., 2019; Malik et al., 2021b). We therefore assessed whether pan-neuronal disruption of DAP5 had any impact on survival of (CGG)90-EGFP expressing flies. Knockdown of DAP5 enhanced lifespan of (CGG)90-EGFP expressing flies (Fig. 4C), although the overall impact was not robust, we observed a significant extension of lifespan. Together, these results suggest that DAP5 modifies CGG repeat-associated toxicity in *Drosophila* possibly through altering FMRpolyG protein accumulation.

Together, these results suggest that DAP5 modifies CGG repeat-associated toxicity in *Drosophila* at least in part through altering FMRpolyG protein accumulation.

### 2.5. EIF2β and EIF3G modulate both canonical and RAN translation in cell-based reporters

**Fig. 5.**
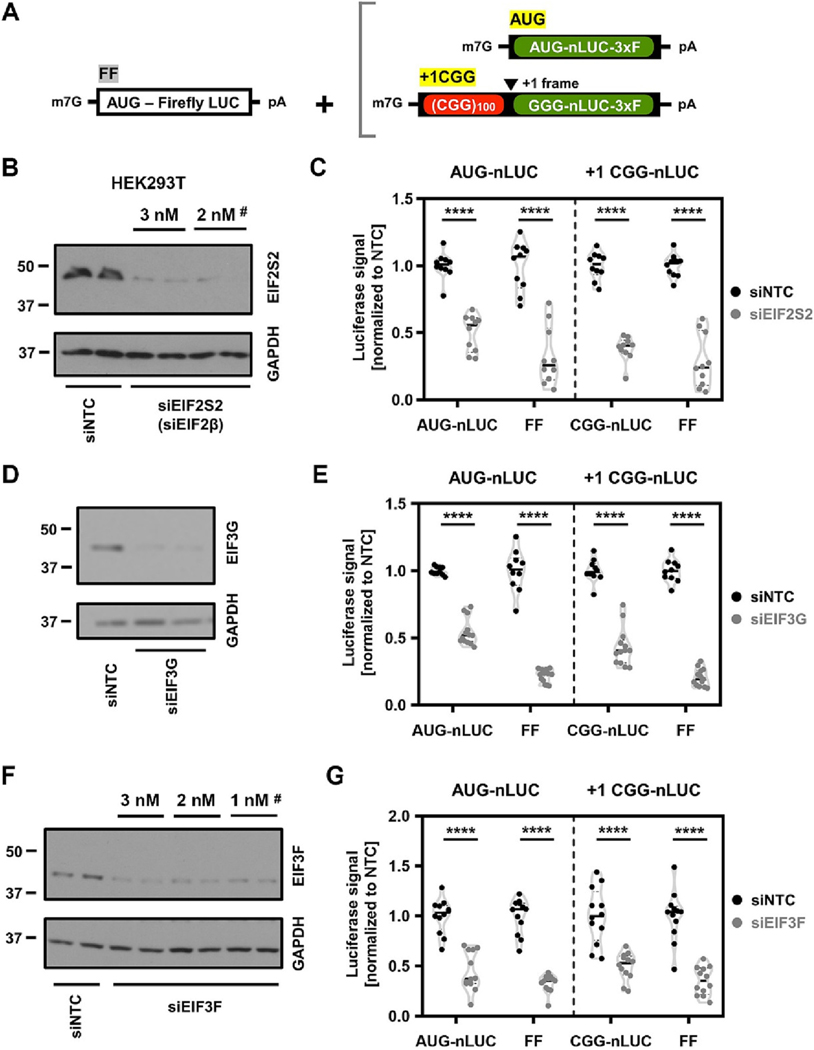
eIF2β and eIF3G knockdown in human cells suppresses both general and RAN translation. (A) Schematic of AUG initiated [AUG-nLUC-3xFLAG] and + 1CGG RAN translation [+1CGG (100)-nLuc-3xFLAG] initiated reporters along with AUG-Firefly (FF) transfection control reporter. (B) Representative western blot shows confirmation of EIF2S2 (eIF2β) knockdown. GAPDH serves as a loading control. ^#^ Denotes the siRNA concentration used in the following experiments. (C) Relative expression of AUG-nLUC and + 1 CGG (100)-nLUC reporters along with co-transfected AUG-FF reporters following knockdown of EIF2S2. (D) Western blot confirmation of EIF3G knockdown. GAPDH is used as a loading control. (E) Relative expression of AUG-nLUC and + 1 (CGG)100-nLUC reporters along with co-transfected AUG-FF reporters following knockdown of EIF3G. (F) Western blot confirmation of EIF3F knockdown. GAPDH is used as a loading control. (G) Relative expression of AUG-nLUC and + 1 (CGG)100-nLUC reporters along with co-transfected AUG-FF reporters following siRNA-mediated knockdown of EIF3F. Statistical analysis: (C, E and G) Two-way ANOVA with Sidak’s multiple comparison test, (*n* = 9–12/condition). **** P < 0.0001.

We next assessed whether any of the modifying initiation factors identified in flies also play a role in RAN translation in human cells. To this end, we used previously characterized luciferase-based reporters consisting of a 3xFLAG-tagged nanoluciferase downstream of the CGG repeats (+1CGG-nLUC-3xF) (Kearse et al., 2016; Malik et al., 2021b). We first asked if any of the canonical translation initiation factors that modified fly phenotypes in our screen, namely - EIF2β, EIF3G and EIF3F-might facilitate RAN translation of cellular reporters. We used AUG-driven 3xFLAG-tagged nanoluciferase (AUG-nLUC-3xF) as a control for canonical translation and firefly luciferase as a transfection control (Fig. 5A). During canonical translation initiation, EIF2β first binds with other initiation factors including EIF1, EIF5, and EIF3. These interactions are destabilized when EIF2β is mutated. siRNA knockdown of EIF2β robustly reduced the expression of plasmid-based +1CGG RAN reporter as well as AUG-driven canonical reporters in HEK293T cells (Fig. 5B–C). Likewise, siRNA knockdown of EIF3G also robustly reduced the expression of both +1CGG RAN reporter and AUG-driven canonical reporter in HEK293T cells (Fig. 5D–E). Notably, EIF3F was previously identified as a potent modifier of RAN translation for multiple repeats (Ayhan et al., 2018). In our hands, knockdown of EIF3F inhibited both +1CGG RAN reporter and AUG-driven canonical reporter translation (Fig. 5F–G), indicating EIF3F may have a general role in translation initiation of both canonical and + 1CGG RAN reporters. Altogether, these results suggest initiation factors that modify rough eye phenotype in *Drosophila* may not have a specific role in +1CGG RAN translation in cells.

### 2.6. DAP5 has cell type-specific effects on + 1CGG RAN translation

We next asked if DAP5 knockdown can inhibit RAN translation of our cellular reporters. A recent study focusing on the translation of upstream open reading frames (uORF) of GGGGCC repeat expansion associated with C9 ALS/FTD found that DAP5 enhanced uORF-like translation of GGGGCC repeats but inhibited downstream cap-independent translation of GGGGCC expansions (van ‘t Spijker et al., 2022). They also found that the knockdown of DAP5 inhibited AUG-driven firefly luciferase translation. Consistent with this earlier work, DAP5 knockdown significantly inhibited plasmid-based AUG-driven firefly (AUG-FF) luciferase expression in HEK293T cells (Supplementary Fig. 3A–B). However, we did not observe any effect of DAP5 knockdown on AUG-driven nano-luciferase (AUG-nLUC) expression (Supplementary Fig. 3C), perhaps reflecting differences in the stability of these two luciferases (Kearse et al., 2016).

**Fig. 6.**
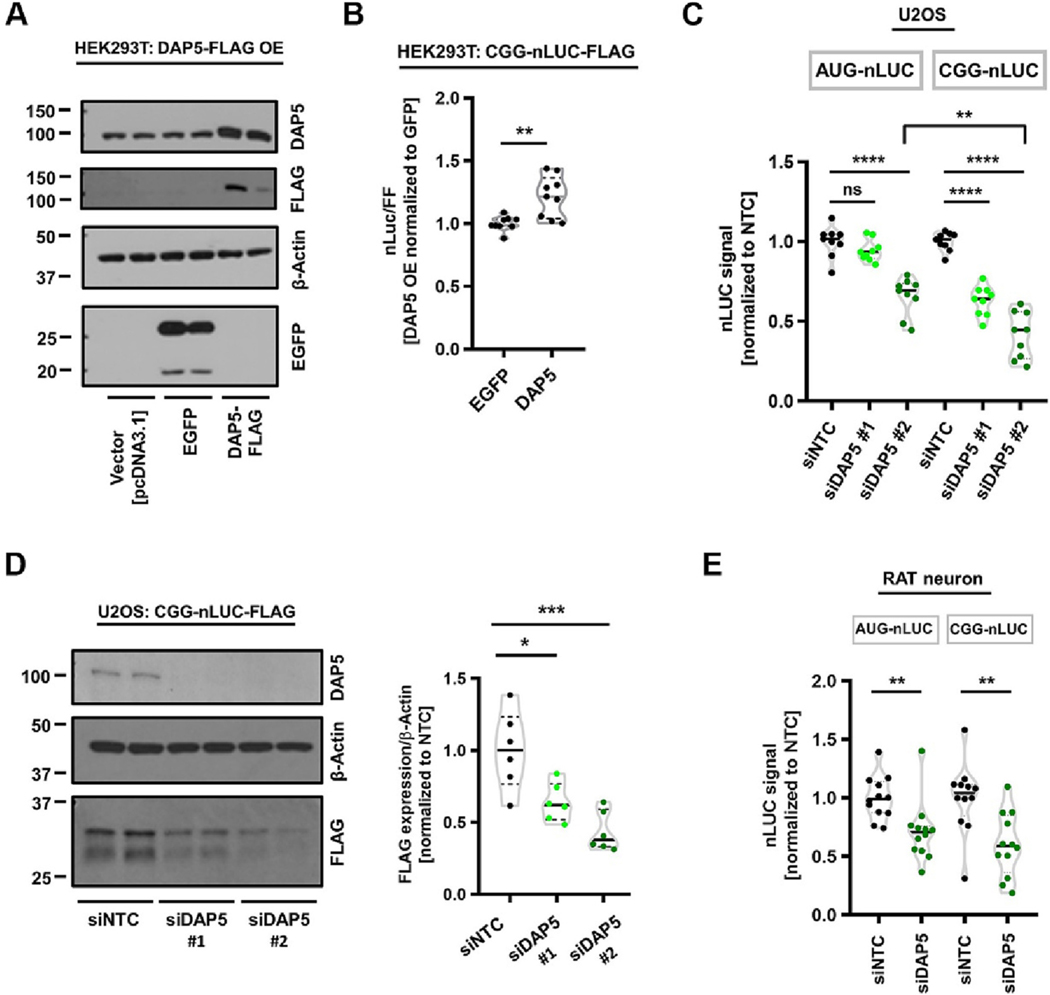
Modulating DAP5 expression impacts CGG RAN translation in a cell-type specific manner. (A) Representative Western blot showing expression of a FLAG epitope-tagged exogenous DAP5 plasmid (DAP5-FLAG) in HEK293T cells. An exogenous EGFP expressing plasmid used for comparison with DAP5 overexpression. β-Actin is used as a loading control. (B) Relative expressions of CGG-nLUC following overexpression of DAP5-FLAG (n = 9/condition) compared to EGFP expression in HEK293T cells. (C) Relative expression of AUG-nLuc and CGG-nLuc reporters in U2OS cells (n = 9/condition) following knockdown of DAP5. (D) Anti-FLAG immunoblots showing effects of DAP5 knockdown on expression of +1CGG (100)-nLuc-3xFLAG RAN translation reporter in U2OS cells. β-Actin is used as a loading control (*n* = 6/conditions). (E) Relative expression of AUG-nLuc and CGG-nLuc reporters in cultured RAT neurons (n = 9/condition) following knockdown of DAP5.

Next, we asked if DAP5 plays any role in RAN translation of CGG repeats in the +1 reading frame. We observed that DAP5 overexpression significantly augmented +1CGG-nLUC-3xF expressions in HEK293T cells compared to a control plasmid (EGFP) overexpression (Fig. 6A–B). In contrast, we did not observe any effect of DAP5 knockdown on +1 CGG nLUC-3xF expression in HEK293Ts (Supplementary Fig. 3D). Together these results indicate that DAP5 is not essential for +1CGG RAN translation, but that its expression level can impact RAN translation in HEK293T cells.

Non-canonical translation, including RAN translation, may be modulated by tissue-specific factors. Therefore, we wondered if DAP5 has any cell-type specific role in regulating RAN translation. To this end, we tested the effects of DAP5 knockdown on RAN translation of CGG repeats in U2OS, RAT hippocampal neurons and SHSY5Y cells. In U2OS cells, DAP5 knockdown significantly and somewhat specifically inhibited RAN translation of CGG repeats in the +1 reading frame (Fig. 6C–D, Supplementary Fig. 4A). However, in SHSY5Y cells, the effects of DAP5 knockdown were non-specific, with effects on both RAN translation and AUG initiated nano-luciferase translation (Supplementary Fig. 4B). As in SHSY5Y cells, DAP5 knockdown inhibited both AUG-driven and RAN translation-driven expression of nano luciferase reporters in transfected Rat hippocampal neurons (Fig. 6E). Together these results suggest DAP5 may have a cell type-specific effects on both canonical and RAN translation.

Previous studies have shown that RAN translation is augmented by cellular stress (Cheng et al., 2018; Green et al., 2017; Westergard et al., 2019). Moreover, RAN translation products enhance cellular stress creating a feed-forward loop, where RAN translated toxic peptides promote more RAN translation (Green et al., 2017). Therefore, we asked if DAP5 knockdown has any effects on cellular stress or RAN translation during such stress events. Although DAP5 has previously been reported to be a part of stress granule components, we did not observe any change in stress granule induction upon DAP5 knockdown (Supplementary Fig. 5A). Moreover, stress-induced enhancement of RAN translation of CGG-nLUC-3XF reporters was unaffected by knockdown of DAP5 (Supplementary Fig. 5B). Finally, we evaluated the role of the proposed interaction between DAP5 and the cap binding factor EIF3D in modulating +1CGG RAN translation in U2OS cells. Knockdown of EIF3D alone significantly decreased RAN translation (Supplementary Fig. 6). While EIF3D and DAP5 double knockdown showed a numerically greater impact on RAN translation compared to EIF3D alone, it did not show a dramatic augmentation in RAN translation inhibition compared to DAP5 knockdown alone (Supplementary Fig. 6), indicating EIF3D and DAP5 may function together but that DAP5 is the key factor. Altogether, our data suggest DAP5 has relatively modest and cell type-specific effects on CGG repeat associated RAN translation in mammalian cell-based reporter systems.

## 3. Discussion

GC-rich repeat expansions that cause FXTAS and related neurodegenerative disorders support Repeat Associated Non-AUG initiated translation of toxic peptides (Todd et al., 2013; Zu et al., 2011). As factors required for RAN translation may differ from those required for canonical translation initiation, identification of RAN translation-specific factors could reveal viable therapeutic targets for a class of currently untreatable disorders. Here, we evaluated the role of EIF4G homologs in modulating CGG RAN translation and CGG-repeat associated toxicity in a *Drosophila* model of FXTAS. We identified DAP5 and its interacting initiation factors - EIF2β, EIF3F and EIF3G- as suppressors of CGG repeat-triggered toxicity in a fly model of FXTAS. However, modulating DAP5 in mammalian cell-based reporter assays elicited only modest and cell-type specific effects on RAN translation, with additional effects in some contexts on a canonical AUG initiated reporter. These studies suggest that further work will be required to fully understand the role of DAP5 in FXTAS pathogenesis.

DAP5 was initially discovered as a 97-kDA protein homolog of EIF4G1 that was required for IRES-dependent initiation on cellular mRNAs (Imataka et al., 1997; Levy-Strumpf et al., 1997; Yamanaka et al., 1997). Unlike EIF4G1 and 4G II, DAP5 lacks the EIF4E-binding domain and immunoprecipitation with m^7^GpppG conjugated Sepharose beads fails to pull-down DAP5 (Liberman et al., 2015). However, recent genome-wide studies suggest that DAP5 can facilitate cap-dependent translation (de la Parra et al., 2018; Weber et al., 2022), especially from transcripts containing a uORF. As such, DAP5 is an intriguing candidate modifier for RAN translation at CGG repeats, which shares many features with uORF-mediated translation initiation and is largely a cap-dependent process both *in vitro* and in transfected cells (Kearse et al., 2016). In this study, DAP5 knockdown significantly modified CGG repeat-associated toxicity in a *Drosophila* disease model, at least in part, through inhibition of neuronal +1CGG RAN-translated product generation. Moreover, targeted knockdown of initiation factors previously shown to preferentially interact with DAP5 also modified CGG repeat toxicity in *Drosophila,* further linking this complex to CGG RAN translation. However, these robust effects were different from what we observed in our mammalian cell-based reporter assays. The effects DAP5 knockdown on both AUG-initiated translation and RAN translation were different across mammalian cell types, with no significant effects observed in HEK293T cells but a RAN-translation selective effect when the same experiment was performed in U2OS cells (Fig. 6). There were also differences in the impact of DAP5 knockdown on Firefly luciferase versus nanoluciferase in mammalian cells (Supplemental Fig. 3), which complicated our ability to account for potential differences in transfection efficiency.

A recent large-scale modifier study using a (GGGGCC)x49 repeat-containing fly model of C9 ALS/FTD found that DAP5 knockdown mildly suppressed GGGGCC repeat-associated toxicity (Goodman et al., 2019). However, in a knock-in HeLa cell reporter system expressing GGGGCC repeats with upstream C9orf72 sequences (including exon 1 sequence that is not present in fly models), DAP5 knockdown inhibited a putative uORF translation above the repeats, and enhanced RAN translation of GGGGCC repeats downstream (van’t Spijker et al., 2022). The difference in DAP5 effects on RAN translation between GGGGCC and CGG repeats and between flies and cell-based assays for GGGGCC repeats likely reflect differences in the nature of the transcripts. For example, CGG RAN translation in HEK293 cells is largely cap-dependent while the assays performed by van’t Spijker and colleagues were largely cap-independent (van’t Spijker et al., 2022). Several recent studies have shown that DAP5 knockdown affects only a subset of transcripts, suggesting a transcript-specificity of DAP5-mediated initiation (de la Parra et al., 2018; Sugiyama et al., 2017; Weber et al., 2022). Moreover, FMRpolyG production is highly dependent on specific near-cognate codons in our experimental system, which may make it distinct from the within-repeat initiation observed at GGGGCC repeats. Taken in this broader context, our findings suggest that DAP5 and its interacting partners likely have different effects across cellular, sequence and species contexts that complicate a simple “one-size fits all” role in RAN translational initiation. A similar outcome was observed for the knockdown of initiation factors EIF4B and EIF4H that showed a significant rescue of CGG toxicity in flies, but a less specific inhibitory effect on both AUG-initiated translation and RAN translation (Linsalata et al., 2019) but a more specific impact on RAN translation from GGGGCC repeats in a separate *Drosophila* model (Goodman et al., 2019). Finally, DAP5 is known to play a role in regulating the translation of apoptosis-associated transcripts. Therefore, the observed effect of DAP5 knockdown in flies may, in part, can be due to the mitigation of CGG repeat toxicity-induced apoptosis (Handa et al., 2005; Hukema et al., 2014).

EIF3 is a large multimeric complex that is essential for both cap-dependent and independent translation initiation. We found that knocking down any of three different EIF3 subunits- EIF3D, EIF3F and EIF3G significantly suppressed CGG repeat-associated toxicity in flies. EIF3F has previously been shown to modulate RAN translation in cellular reporters (Ayhan et al., 2018). In our hands, EIF3F knockdown also affected AUG-initiated luciferase reporter synthesis, which is consistent with the known canonical role of EIF3 in general translation (Fig. 5). EIF3G is a component of the entry channel arm of EIF3 and it directly interacts with the CGG RNA (Malik et al., 2021b). Like EIF3F, EIF3G knockdown suppressed synthesis of both canonical translation and RAN translational reporters. EIF3D was recently identified as a novel cap-binding factor necessary for translation of specific subset of RNAs (de la Parra et al., 2018; Lee et al., 2016). As DAP5 has no cap-binding module, EIF3D was recently proposed as a facilitator of the cap-dependent DAP5-mediated translational (de la Parra et al., 2018; Lee et al., 2016). While our EIF3D-DAP5 double knockdown data are consistent with the notion that DAP5 may work in partnership with EIF3D, the exact mode of this interaction and requirement of specific domain in respect to RAN translation initiation needs further studies.

In summary, our data suggest a potential role of DAP5 in RAN translation at CGG repeats that may contribute to neurodegeneration, but with significant caveats related to cell-type, species, and, perhaps, repeat sequence context. CGG trinucleotide repeat expansions outside of the *FMR1* loci have recently emerged as a cause of multiple degenerative and developmental disorders (Zhou et al., 2022), with clinical phenotypes ranging from distal myopathy to motor neuron disease to leukoencephalopathy. While this project focuses on a single sequence context, variations in CGG repeat associated translation will likely be relevant to these observed variations in disease phenotypes. Embracing rather than rejecting this complexity will likely yield both novel mechanistic insights and critical clues needed to develop effective therapies for these currently untreatable disorders.

Statistical analysis: (A, E) Two-tailed Student’s t-test with Welch’s correction. (C-D) One-way ANOVA with Dunnett’s multiple comparison test. * P < 0.05, ** P < 0.01, *** P < 0.001, **** P < 0.0001 and ns = non-significant.

## 4. Methods

### 4.1. Antibodies

The following antibodies were used for western blots as specified for dilution in 5% non-fat milk; FLAG-M2 at 1:1000 dilution (mouse, Sigma F1804), 1:2500 β-Actin (mouse, Sigma A1978), 1:2500 GAPDH 6C5 (mouse, Santa Cruz sc32233), 1:1000 DAP5 (rabbit, CST 5169S), 1:1000 EIF3G (rabbit, Bethyl Laboratories, Inc. A301–757A-T), 1:1000 EIF3F (rabbit, Bethyl Laboratories, Inc. A303–005A-T), 1:1000 EIF2β (rabbit, Protein Tech 10,227–1-AP), 1:1000 GFP (mouse, Roche/Sigma 11,814,460,001). HRP-conjugated goat-anti-mouse (115–035–146) or goat-anti-rabbit (111–035–144) secondary antibodies (Jackson ImmunoResearch Laboratories) were used at a 1:10,000 dilution in 5% non-fat dry milk.

The following antibodies were used for immunocytochemistry as specified; 1:500 DAP5 (rabbit, CST 5169S), 1:200 G3BP (mouse, BD Transduction Laboratories 23/G3BP) in 5% normal goat serum (NGS). Secondary goat-anti-mouse Alexa Fluor 488 (A-11029) and goat-anti-rabbit Alexa Fluor 647 (A-21244) from Life Technologies were applied at 1:500.

### 4.2. Drosophila lines and eye phenotyping

All *Drosophila* lines used in this study and their sources are listed in Supplementary Table 1.

All flies were maintained on SY10 food at 25 °C. CGG repeat size was confirmed by PCR using published (C and F) primers, western blots of RAN products and established protocols (Todd et al., 2013). For rough eye phenotyping, virgin female flies expressing the UAS-FMR1 (CGG)90-EGFP reporter and a GMR-GAL4 driver were crossed with male flies carrying a germline mutation or an UAS-driven siRNA targeting the candidate gene. Rough eye phenotypes of resulting F1 progenies, carrying both CGG repeats and the modifier, were evaluated 1–2 days post eclosion using Leica M125 stereomicroscope. Minimum 20 flies (male and females) from two independent crosses were scored in following criteria - (i) abnormal orientation of the bristles, (ii) presence of supernumerary bristles, (iii) ommatidial fusion and disarray, (iv) presence of necrotic tissues, and (v) collapse/shrinkage of the eye. Possible scores were given for each criterion in a scale of 0–5: 0 (no abnormality), 1 (mere presence of the abnormality, but <5%), 3 (abnormality affecting >5% of the total eye), and 5 (abnormality affecting >50% of the total eye).

For survival experiments, virgin female flies carrying an ElaV-GAL4 GS-driver were crossed with male flies containing CGG repeats and a UAS-siRNA transgene against DAP5. F1 progenies were collected 1–2 days post eclosion and placed on SY10 food containing 200 μM RU486. Each tube containing ~20 flies (total > 100 flies per condition; equal male and females) were housed at 29 °C and were flipped onto fresh RU486 supplemented SY10 every 48 hours. Number of deaths was recorded during each flip and was subsequently plotted using GraphPad Prism.

### 4.3. Drosophila external eye fluorescent measurement

Eye inclusion measurement, external eye fluorescent was measured essentially as described earlier. Fly crosses were performed as described above - GMR-GAL4 virgin females were crossed to male flies containing the UAS-siRNA transgene against DAP5 or a non-targeting control at 25 °C. Fluorescent images were taken at 1–2 days post eclosion using a Leica M125 stereomicroscope and a Leica DFC425 digital camera with GFP filter. All images were taken at the same exposure. Raw GFP images were converted to grey scale and total intensity of inclusions was measured using ImageJ as described earlier.

### 4.4. Drosophila Western blot

For immunoblotting of fly material, 1–2 days post-eclosion flies carrying (CGG)90-EGFP and ELAV GS-driver with the UAS-siRNA transgene against DAP5 or a non-targeting control were placed on 200 μM RU486-supplemented SY10 food for 5 days. Flies were kept at 29 °C and fresh RU486-supplemented food was provided every 24 hours. To collect fly tissue materials, flies were homogenized in RIPA buffer supplemented with protease inhibitor (Roche), using a handheld homogenizer at 4 °C. Homogenized fly material was centrifuged at 13,500 g for 10 min at 4 °C to pellet tissue debris. Cleared lysate was removed, mixed with 6 × SDS sample buffer, and boiled at 90 °C for 10 min before running on SDS–PAGE for Western blotting.

### 4.5. Cells, cultured neurons, and reporter assays

HEK293T, U2OS and SHSY5Y cells were purchased from American TypeCulture Collection (ATCC) and cultured in DMEM or DMEM F12 1:1 (Fisher) media supple-mented with fetal bovine serum (Bio-Techne) (v: v). They were maintained at 37 °C with 5% CO2 and, checked for mycoplasma contamination in regular intervals. AUG-nano luciferase (AUG-nLUC-3×Flag) and + 1CGG-nano luciferase (+1CGG-nLUC-3×Flag) reporters used in this study are previously published and well characterized (Kearse et al., 2016; Malik et al., 2021b). FLAG-tagged DAP5 construct (DAP5-FLAG) used for overexpression experiment is also published earlier (Kearse et al., 2019).

For cultured neuron transfections, rat embryonic cortical dissections from E20 rat pups of both sexes were performed as previously described (Malik et al., 2021b; Flores et al., 2016). Dissociated cortical neurons were plated at 0.6 × 10^5^ cells per well on poly-D-lysine-coated 96-well plate in neuronal growth media containing NGM, neurobasal A media, 2% B-27, 1% Glutamax-1 (v:v), and maintained at 37 °C for 4 days before transfection. Neuronal transfections were performed using Lipofectamine^™^ 3000 transfection reagent, following manufacturer protocol.

For luminescence assays, cells were seeded in 96-well plates at a concentration of 1.0 × 10^4^ cells/well in 100 μL media and reverse transfected with siRNAs using Lipofectamine RNAiMax according to manufacturer instructions. Briefly, siRNA and RNAiMAX were diluted in reduced serum media (Opti-MEM), mixed gently, and incubated for 10 min before adding to the cells. Following siRNAs (Thermo Fisher Scientific) were used for knockdown of target genes – DAP5 (siRNA#1 ID: s4588; siRNA#2 ID: HSS103156), EIF2β (siRNA ID: S17002), EIF3G (siRNA ID: s225017) and EIF3F (siRNA ID: s16501) were used for KD. Subsequently, 48 hours post siRNA transfection, reporter plasmids were transfected using jetPRIME (Polyplus) reagent as described earlier. In brief, 25 ng nLuc reporter plasmid and 25 ng pGL4.13 Firefly luciferase reporter plasmid were mixed with the jetPRIME buffer at a ratio of 2:1 (jetPRIME: DNA) for 10 mins at room temperature, and then added to the cells. For stress induction, 2 μM TG or vehicle control (DMSO) was added to HEK293T cells 19 hours post reporter transfection for 5 hours. Luminescence was measured using the ONE-Glo Luciferase Assay System (Promega) on a GloMax Microplate Luminometer.

For western blots, cells were seeded in 24-well plates. siRNA and reporter transfections were performed using RNAiMaX and jetPrime, respectively, as described above. 24 hours after the reporter plasmid transfection, cells were lysed in 300 μL cold RIPA buffer supplemented with protease inhibitor (cOmpleteTMMini, Sigma) for 30 min at 4 °C with occasional vortexing. Lysates were cleared by centrifugation at 4 °C. Supernatant was collected, mixed with 6 × SDS sample buffer and boiled at 90 °C for10 min before running on SDS–PAGE. Gels were transferred to PVDF membranes for 2.5 hours at 320 mAmps and 4 °C. Alternatively, if needed, overnight transfer was performed at 40 V. Transferred membrane was blocked with 5% non-fat milk and probed with primary antibodies diluted in 5% non-fat milk. Western band intensities were measured using ImageJ and plotted using GraphPad Prism.

### 4.6. Stress granule analysis

U2OS cells were seeded in 8-well chamber slides at a concentration of 2.0 × 10^4^ cells/well in 250 μL media and reverse transfected with siRNAs using Lipofectamine RNAiMax according to manufacturer instructions. 72 h after transfection cells were washed thrice with PBS containing 1 mM MgCl2 and 0.1 mM CaCl2 (PBS-MC) and fixed in 4% paraformaldehyde (PFA) in PBS-MC for 15min at room temperature. Next, cells were washed thrice again with PBS-MC and subsequently permeabilized with 0.1% TritonX in PBS-MC for 5min at room temperature. Then cells were blocked with 5% normal goat serum (NGS) in 0.1% Triton-PBS-MC for 1 hour at room temperature before incubating with primary antibodies diluted in 5% NGS, 0.1% TritonX PBS-MC, overnight at 4 °C. Next day, primary antibodies were removed, cells were washed 3x in PBS-MC, and then incubated with Alexa-Fluor secondary antibodies for 1h at room temperature in the dark. Finally, cells were washed thrice with PBS-MC and coverslips were applied with ProLong Gold Antifade with DAPI (ThermoFisher). 4–5 fields per condition were imaged at 20× and 40× objectives using Olympus IX71 fluorescence microscope. For stress granule induction, cells were treated with 2 μM TG for 2 h. For image analysis, raw signals from the stress granule channel were converted to CMYK color models using the ImageJ program. After background subtraction, stress granule signal (particles) from the images measured in binary mode. 4–5 views per each condition with a total number of 761–1010 cells were quantified and the graph was plotted using GraphPad Prism.

## Supplementary Material

Suppl material

## Data Availability

No Genbank datasets were generated as part of this study. Raw images and complete western blots used in this study are included as an appendix. Further information is available upon request.
