# Supplementary material for: Dissecting the roles of EIF4G homologs reveals DAP5 as a modifier of CGG repeat-associated toxicity in a Drosophila model of FXTAS
Source: Neurobiol Dis. Author manuscript; Available in PMC 2024 Jun 4. (PMC11149892; doi:10.1016/j.nbd.2023.106212)
Supplement: Suppl material [file NIHMS1989002-supplement-Suppl_material.docx]

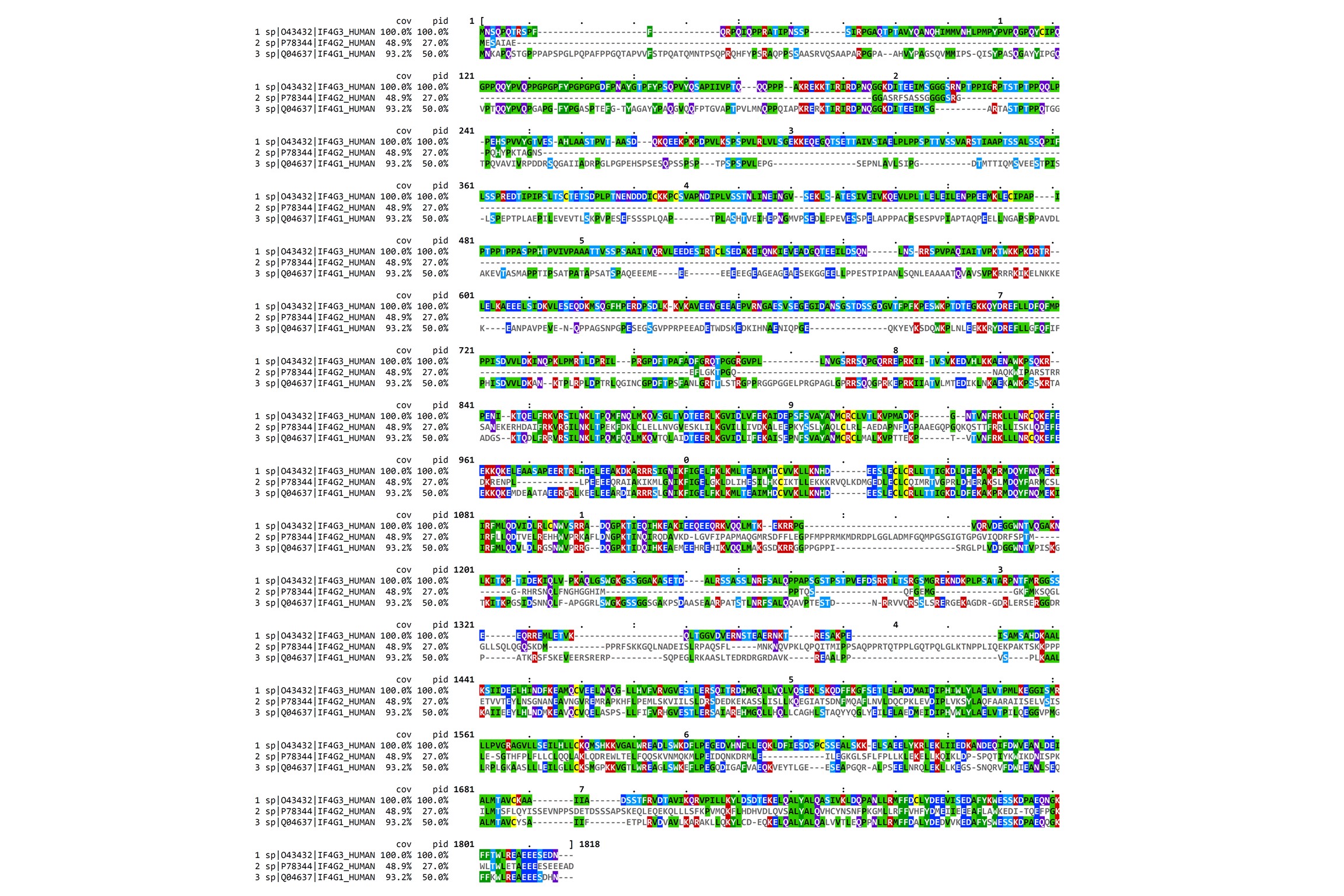


**Supplementary Figure 1. Sequence alignment between eIF4Gs**

Protein sequences of human EIF4Gs were aligned and displayed using Clustal Omega, demonstrating the sequence conservation.

**A**


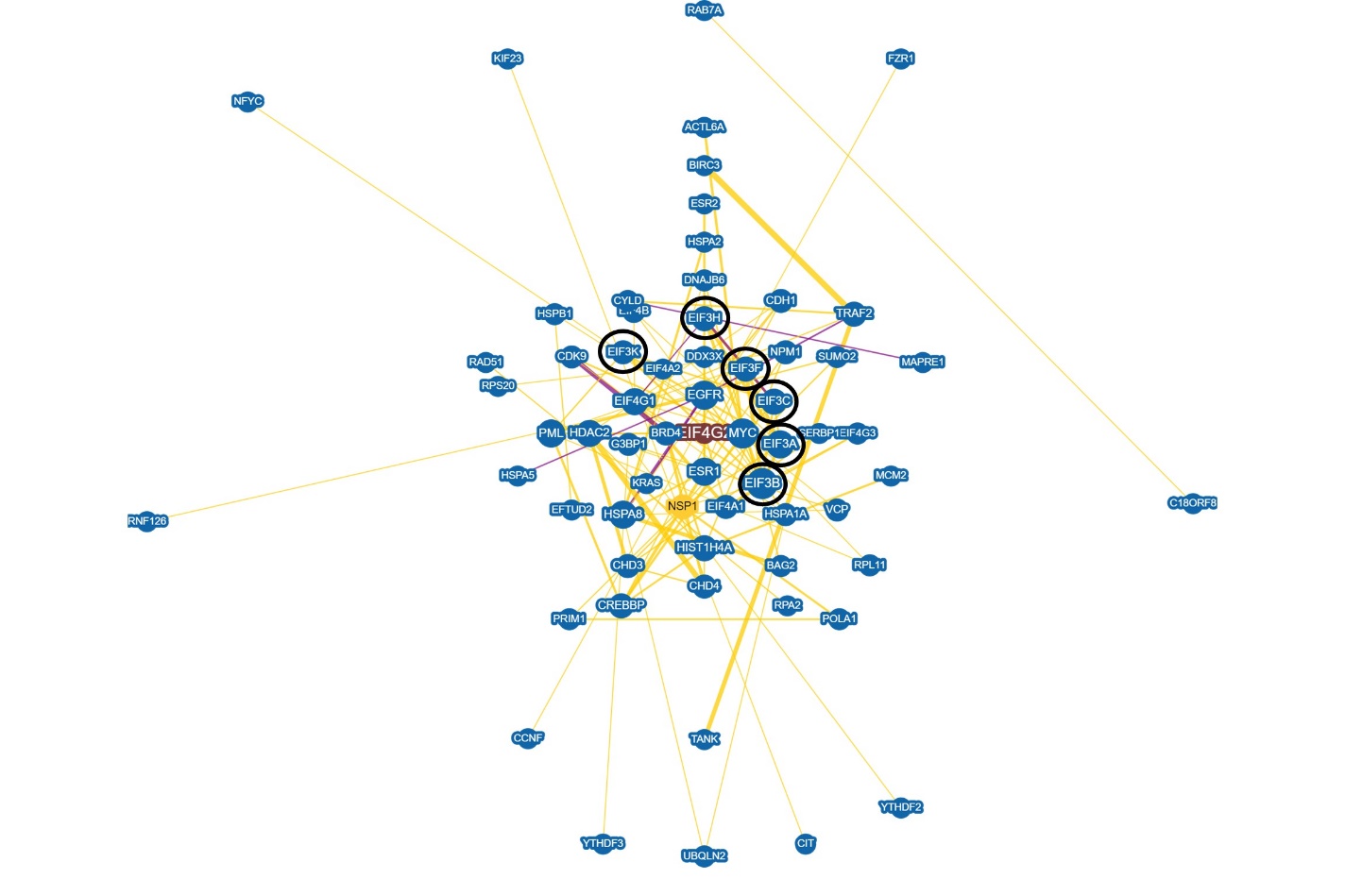


**B**


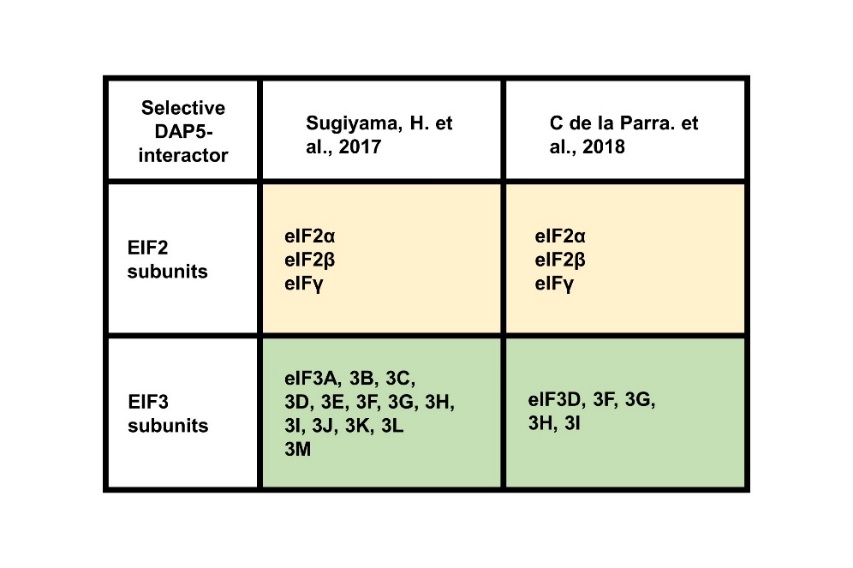


**Supplementary Figure 2. DAP5 interacts with additional eIF2 and eIF3 subunits compared to EIF4G1. (A)** Predicted protein interactions of DAP5/EIF4G2 shows enrichment of multiple EIF3s (black circled). **(B)** Comparative analysis of selective DAP5-interacting factors over EIF4G1/II from two independent studies show enrichment of specific eIF2 and eIF3 subunits.

**
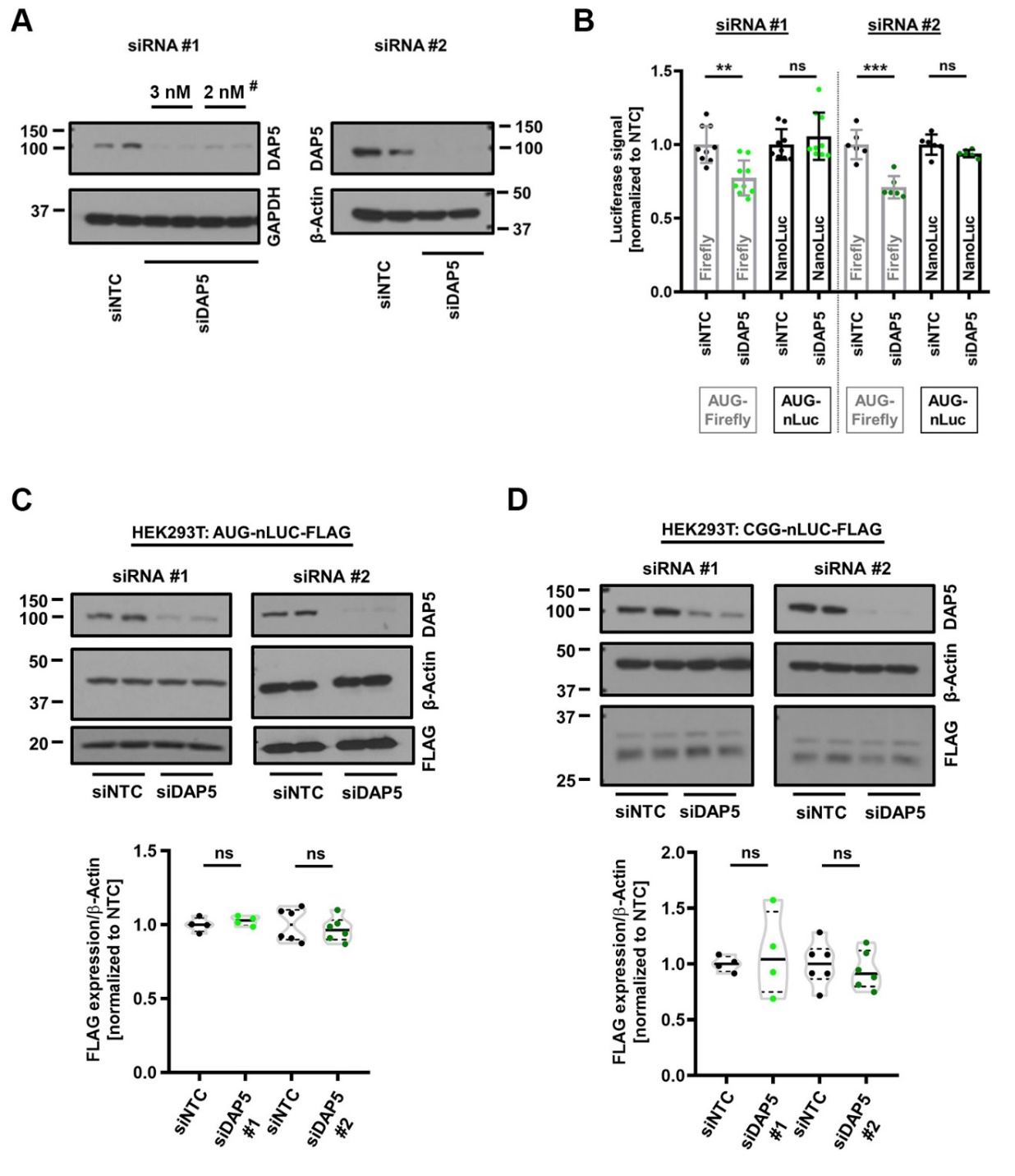
**

**Supplementary Figure 3. DAP5 knockdown inhibits AUG-FF but not AUG-nanoluciferase expression in HEK293T cells.**

**(A)** Representative western blots showing confirmation of DAP5 knockdown for two different siRNAs. GAPDH and β-actin are used as loading controls for siRNA – 1 and siRNA – 2, respectively. (2 nM) ^#^ Denotes the siRNA concentration used in the following experiments. **(B)** Relative expressions of AUG-nLUC along with co-transfected AUG-FF reporters following knockdown of DAP5 (n = 6-9/condition). **(C)** Anti-FLAG immunoblots showing effects of DAP5 knockdown on the expression of AUG-nLUC-3xFLAG reporter in HEK293T cells (top). β-Actin is used as a loading control. (bottom) Quantitation of AUG-nLUC-3xFLAG expression normalized to control siRNA (n = 4-6/conditions). **(D)** Anti-FLAG immunoblots showing effects of DAP5 knockdown on the expression of +1CGG (100)-nLuc-3xFLAG RAN translation reporter in HEK293T cells (top). β-Actin is used as a loading control. (bottom) Quantitation of FLAG expression normalized to control siRNA (n = 4-6/conditions).

Statistical analysis: Two-tailed Student’s t-test with Welch’s correction. ns= not significant, ** P<0.01, *** P<0.001.**
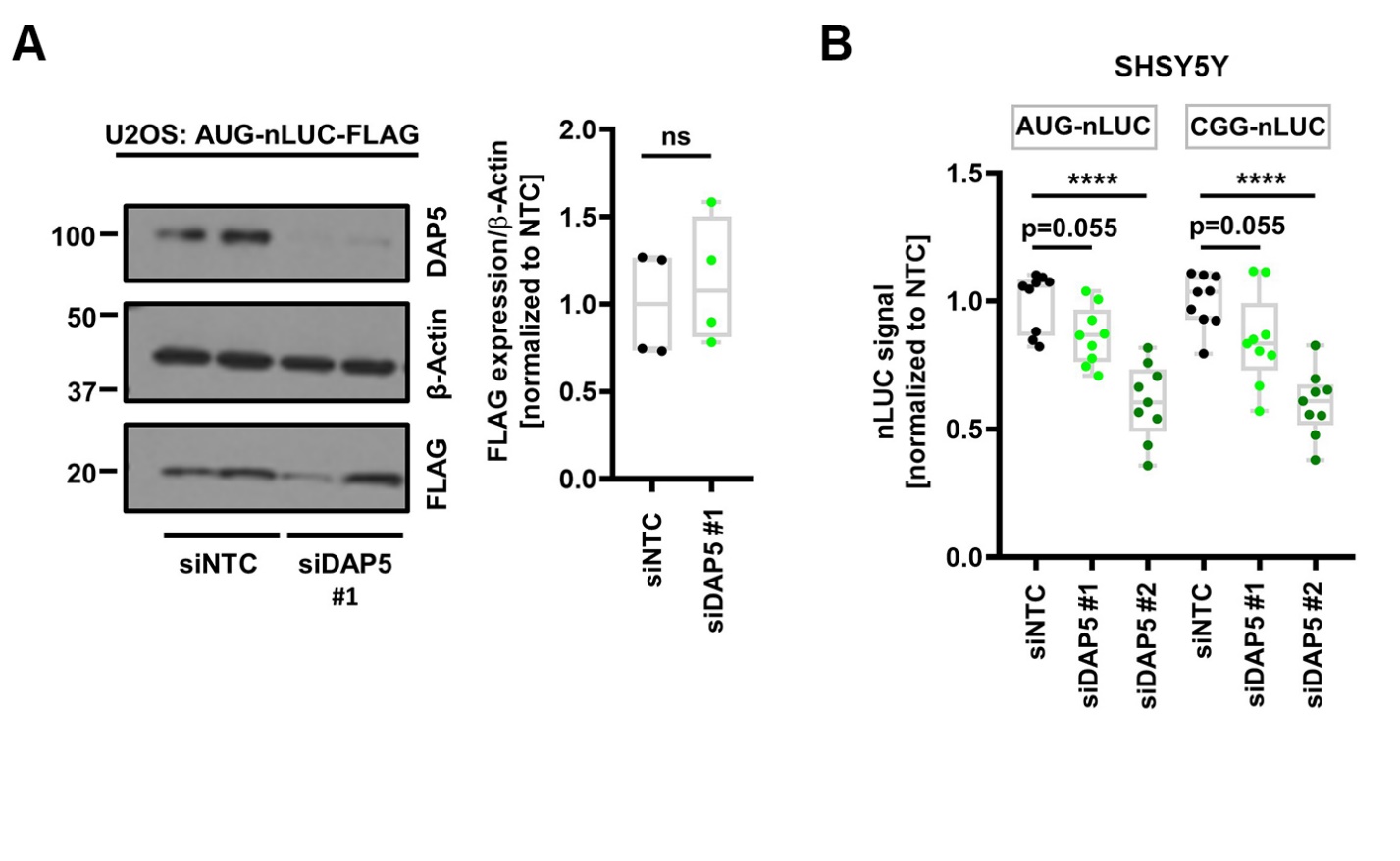
Supplementary Figure 4. Effects of DAP5 knockdown on nano luciferase and AUG-FF reporter expression in U2OS and SHSY5Y cells (A)** Anti-FLAG immunoblots showing effects of DAP5 knockdown on the expression of AUG-nLUC-3xFLAG reporter in U2OS cells (n = 4/conditions). β-Actin is used as a loading control. Two-tailed Student’s t-test with Welch’s correction, ns = non-significant. **(C)** Relative expression of AUG-nLuc and CGG-nLuc reporters in SHSY5Y cells (n = 9/condition) following knockdown of DAP5. One-way ANOVA with Dunnett’s multiple comparison test. **** P<0.0001


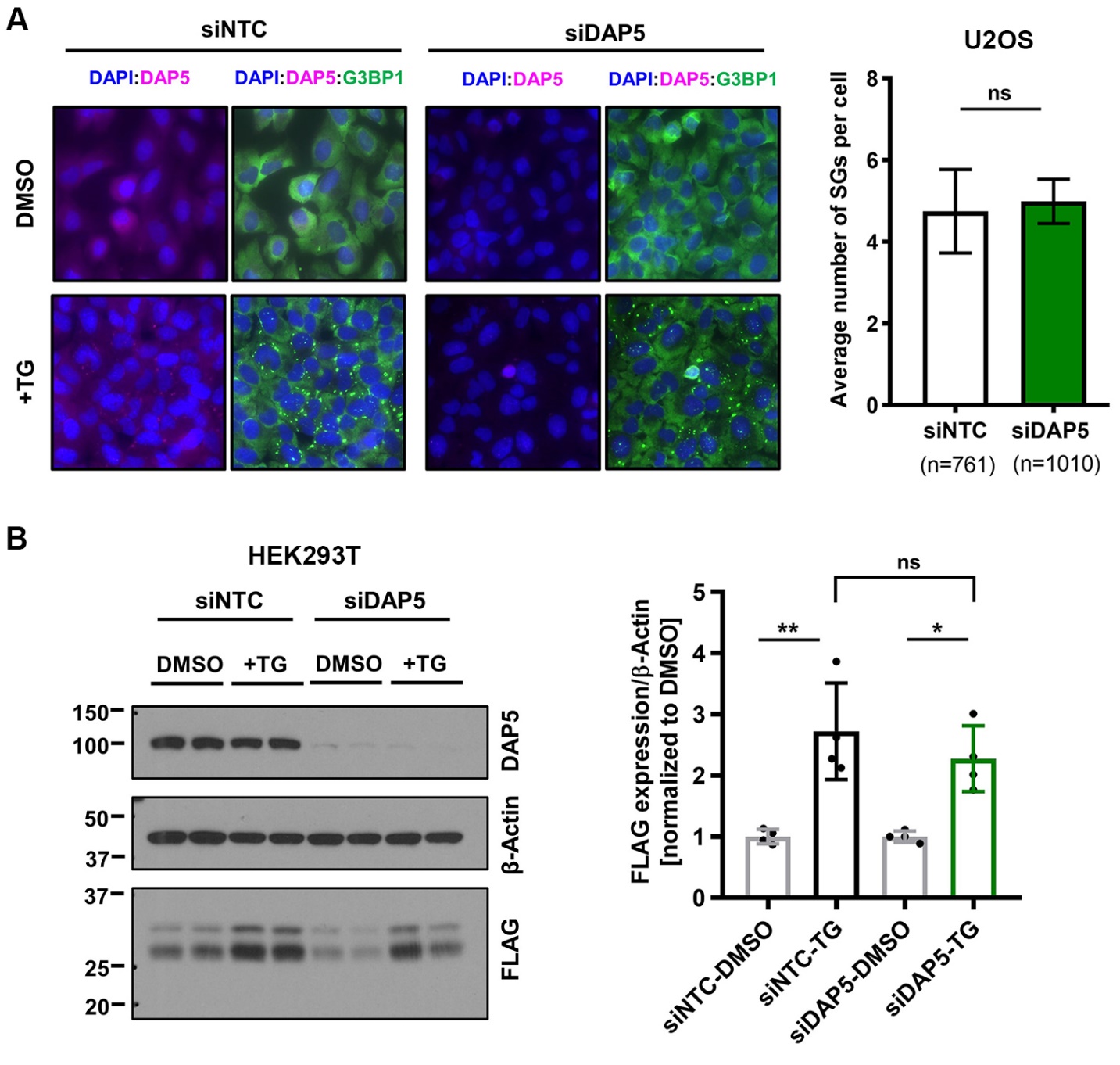


**Supplementary Figure 5. DAP5 knockdown does not alter stress granule formation or RAN translation enhancement by cellular stress (A)** Immunofluorescent images of U2OS cells treated with DMSO or 2 µM thapsigargin (TG). Comparison of stress granule induction by oxidative stress in presence or absence of DAP5. (left) Quantification of average number of SGs per cell (total SGs/total cell number per view). Statistical analysis: Two-tailed Student’s t-test with Welch’s correction, ns = non-significant. **(B)** Expression of +1CGG‐nLuc‐3xFLAG reporters in HEK293T cells treated with 2 μM TG in presence or absence of DAP5, analyzed by immunoblot (n = 4). (left) Quantitation of FLAG expression normalized to vehicle (DMSO) treatment. Statistical analysis: One-way ANOVA with Bonferroni correction for multiple comparison test. * P<0.05, ** P<0.01, and ns = non-significant.


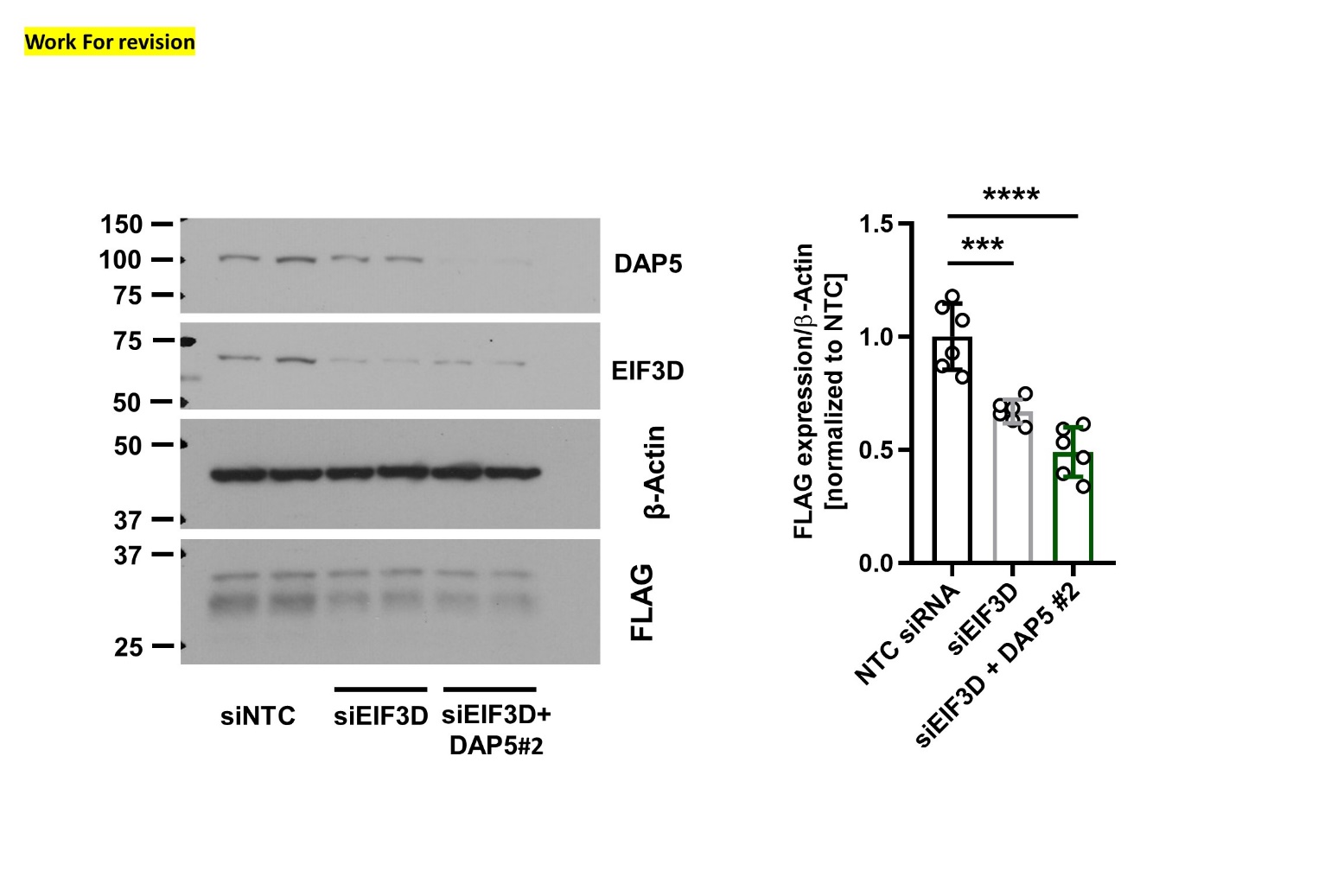


**Supplementary Figure 6. Modulating EIF3D expression alone or conjunction with DAP5 impacts CGG RAN translation in U2OS cells.** Anti-FLAG immunoblots showing effects of EIF3D single and EIF3D-DPA5 double knockdown on expression of +1CGG (100)-nLuc-3xFLAG RAN translation reporter in U2OS cells. β-Actin is used as a loading control (n = 6/conditions). Statistical analysis: One-way ANOVA with Dunnett’s multiple comparison test. *** P<0.001, **** P<0.0001

**Supplementary Table 1. List of *Drosophila* lines used in this study**

| **Modifier Fly Stock** | **Source** | **Stock number** |
| --- | --- | --- |
| EIF4G1 #1 | BDSC | 33049 (UAS RNAi) |
| EIF4G1 #2 | BDSC | 82960 (UAS RNAi) |
| EIF4GII #1 | BDSC | 41963 (UAS RNAi) |
| EIF4GII #2 | BDSC | 35809 (UAS RNAi) |
| DAP5/NAT1 #1 | BDSC | 27302 (UAS RNAi) |
| DAP5/NAT1 #2 | BDSC | 32357 (UAS RNAi) |
| DAP5/NAT1 #3 | BDSC | 13120 (insertion) |
| EIF2α #1 | BDSC | 44449 (UAS RNAi) |
| EIF2α #2 | BDSC | 80414 (UAS RNAi) |
| EIF2β #1 | BDSC | 53268 (UAS RNAi) |
| EIF2β #2 | BDSC | 17425 (insertion) |
| EIF2γ #1 | BDSC | 33401 (UAS RNAi) |
| EIF2γ #2 | BDSC | 32914 (UAS RNAi) |
| EIF3A | BDSC | 31186 (UAS RNAi) |
| EIF3D1 #1 | BDSC | 20072 (insertion) |
| EIF3D1 #2 | VDRC | 330545 (shRNA) |
| EIF3F | BDSC | 33980 (UAS RNAi) |
| EIF3G | BDSC | 43243 (UAS RNAi) |
| EIF3H #1 | BDSC | 55603 (UAS RNAi) |
| EIF3H #2 | BDSC | 82966 (UAS RNAi) |
| EIF3I #1 | BDSC | 34978 (UAS RNAi) |
| EIF3I #2 | BDSC | 14294 (insertion) |
| EIF3D2 #1 | VDRC | 26637 |
| EIF3D2 #2 | VDRC | 104342 |
| siNTC (non-targeting control, luciferase/LUC) | Tod lab | (UAS RNAi) |
| UAS-FMR1 (CGG)90-EGFP | Tod lab |  |
